# Conduction system pacing using a rotatable connector enabling continuous pacing during lumenless lead deployment: a case report

**DOI:** 10.1093/ehjcr/ytag038

**Published:** 2026-02-03

**Authors:** Fernando Montenegro Sá, Alexandra Castro, Sónia Pereira, Cristina Gavina

**Affiliations:** Cardiology Department, Hospital Pedro Hispano, ULS Matosinhos, Senhora da Hora 4464-513, Portugal; Cardiology Department, Hospital Pedro Hispano, ULS Matosinhos, Senhora da Hora 4464-513, Portugal; Cardiology Department, Hospital Pedro Hispano, ULS Matosinhos, Senhora da Hora 4464-513, Portugal; Cardiology Department, Hospital Pedro Hispano, ULS Matosinhos, Senhora da Hora 4464-513, Portugal

**Keywords:** Conduction system pacing, Lumenless lead, Left bundle branch area pacing, Case report

## Abstract

**Background:**

Conduction system pacing (CSP) is an alternative to right ventricular pacing, providing more physiological ventricular activation. Current guidelines recommend CSP for patients with an expected ventricular pacing burden >20% and mildly reduced left ventricular ejection fraction. However, consistent conduction capture is not always achieved, with success rates of around 80% in the MELOS trial. Lumenless leads (LL) are widely used for CSP, but sustained, real-time pacing and impedance monitoring could enhance procedural control and decrease procedure time. The integration of a tool to overcome this limitation was highlighted as a future development in the 2025 EHRA CSP consensus. We report the first case using this approach.

**Case summary:**

An 80-year-old man with paroxysmal complete atrioventricular block and mildly reduced ejection fraction underwent pacemaker implantation. CSP was selected as the pacing strategy according to current recommendations. A Medtronic® 3830 LL was delivered via a C315His sheath, connected to a Micropace® Onestim-CRM stimulator through the new rotator connector 5944RL. This configuration allowed simultaneous septal penetration and continuous pacing for real-time monitoring of current of injury and continuous impedance analysis. CSP success criteria were achieved—left ventricular activation time 62 ms, broad R′ in V1, ventricular pacing thresholds for CSP 1.3 V @ 0.4 ms, fluoroscopy time 4:19 min, and shorter overall procedure (skin to skin, 50 min).

**Conclusion:**

To our knowledge, this case represents the first reported use of a rotatable connector for CSP lumenless lead deployment, demonstrating the feasibility of continuous pacing with LLs for CSP. Further experience is needed to confirm long-term performance and clinical impact.

Learning points

- A rotatable connector enables continuous pacing and real-time impedance monitoring during lumenless lead deployment.
- Continuous feedback during deployment enhances procedural awareness and may simplify CSP workflow.
- This case demonstrates technical feasibility of this approach as an advancement in CSP practice.

## Introduction

Conduction system pacing (CSP), encompassing His bundle pacing and left bundle branch area pacing, has gained increasing attention as a physiologic pacing strategy compared to conventional right ventricular pacing. Implantation with the Medtronic 3830 lumenless lead via a fixed-curve C315His sheath is the most common approach.^1^ However, successful CSP can be technically challenging.^2^ Lead deployment with lumenless leads can require multiple rotations into the interventricular septum, with the operator mainly relying on fluoroscopy and intermittent pacing, fixation beats, or brief impedance checks to assess septal penetration—final placement depending on acceptable pacing-induced R-wave peak time (RWPT) in lead V6, R′ progression in lead V1, V6-V1 RWPT, and QRS transition during threshold tests.^1–3^

Real-time pacing and impedance monitoring during deployment could enhance procedural control and accuracy, providing immediate feedback on tissue engagement and conduction capture.^3^ Here, we report the first use of a rotatable connector enabling continuous pacing during active fixation of a 3830 lumenless lead.

## Summary figure

**Figure:**
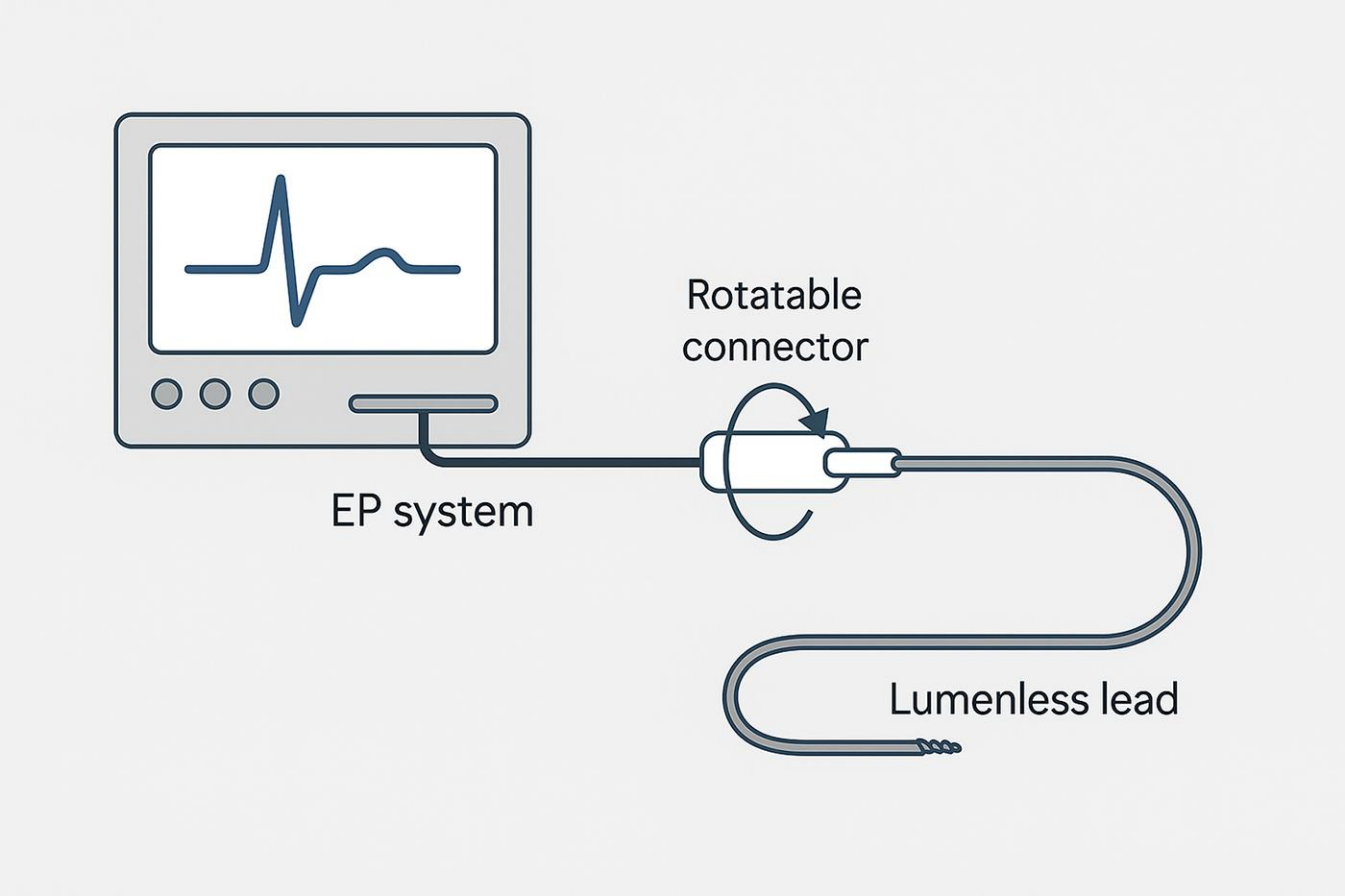
The rotatable connector allows the lumenless lead to connect to an electrophysiology (EP) system with an integrated stimulator, enabling simultaneous mechanical fixation and continuous pacing. The connector maintains electrical continuity during lead rotation, allowing real-time impedance and electrogram monitoring throughout lumenless lead deployment.

## Case presentation

**Figure 1:**
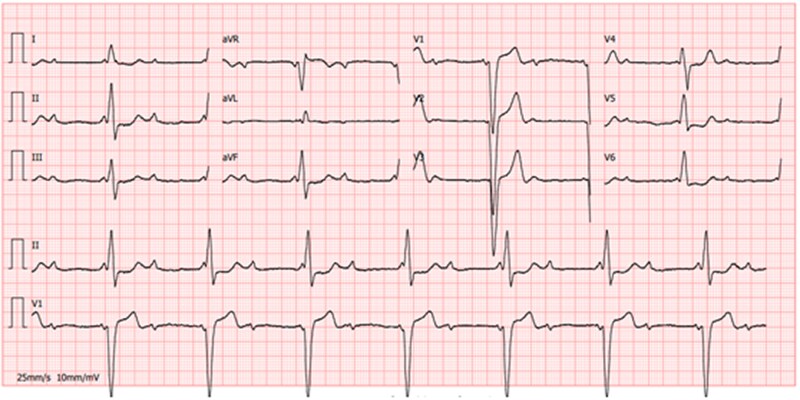
Baseline ECG with complete atrioventricular block and left bundle heart block (baseline QRS 147 ms).

An 80-year-old man with complaints of dizziness was referred for permanent pacemaker implantation due to Mobitz type II atrioventricular block identified in a 24-h Holter and paroxysmal complete AV block, documented on 12-lead ECG—*Figure 1*. His cardiovascular history included hypertension and dyslipidemia. Echocardiography revealed a hypertensive cardiopathy with left ventricular ejection fraction of 49%, without significant valvular disease. Given the anticipated high pacing burden and mildly reduced left ventricular function, CSP was selected to reduce the risk of pacing-induced cardiomyopathy.^3^

**Figure 2:**
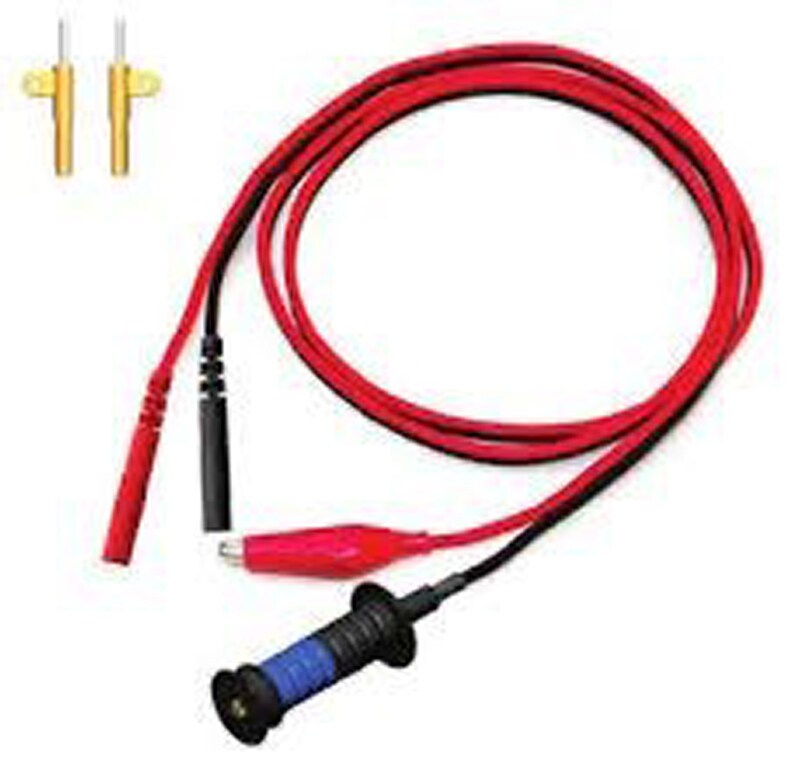
Rotatable connector for the lumenless lead 3830, by Medtronic ©. The cathode cable (black) has a rotatable lead connector at the distal end of the cable. This electrical connection allows real-time continuous pacing and sensing during lead implantation.

**Figure 3:**
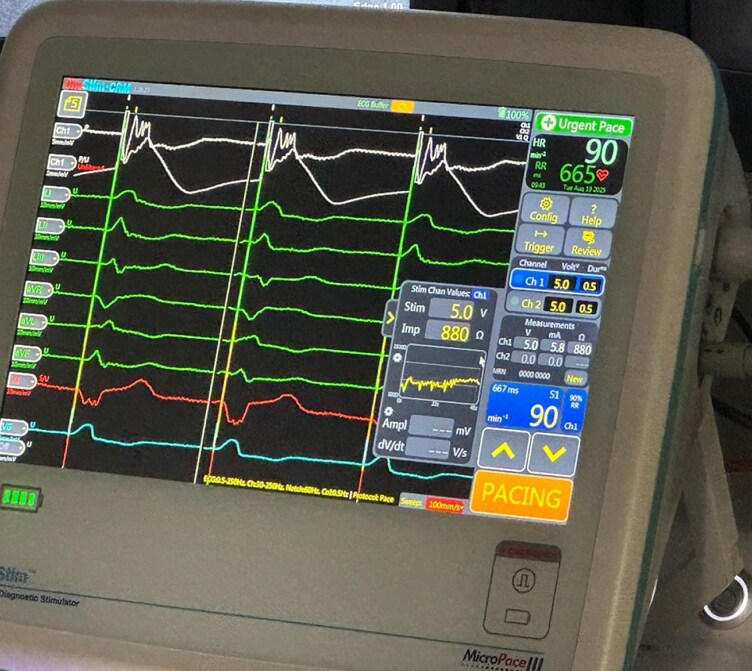
Intracardiac signals revealing a ‘w’ pattern in lead V1 (red), identifying an adequate lead positioning; continuous impedance monitoring can be seen (revealing, at that moment, 880 ohms).

**Figure 4:**
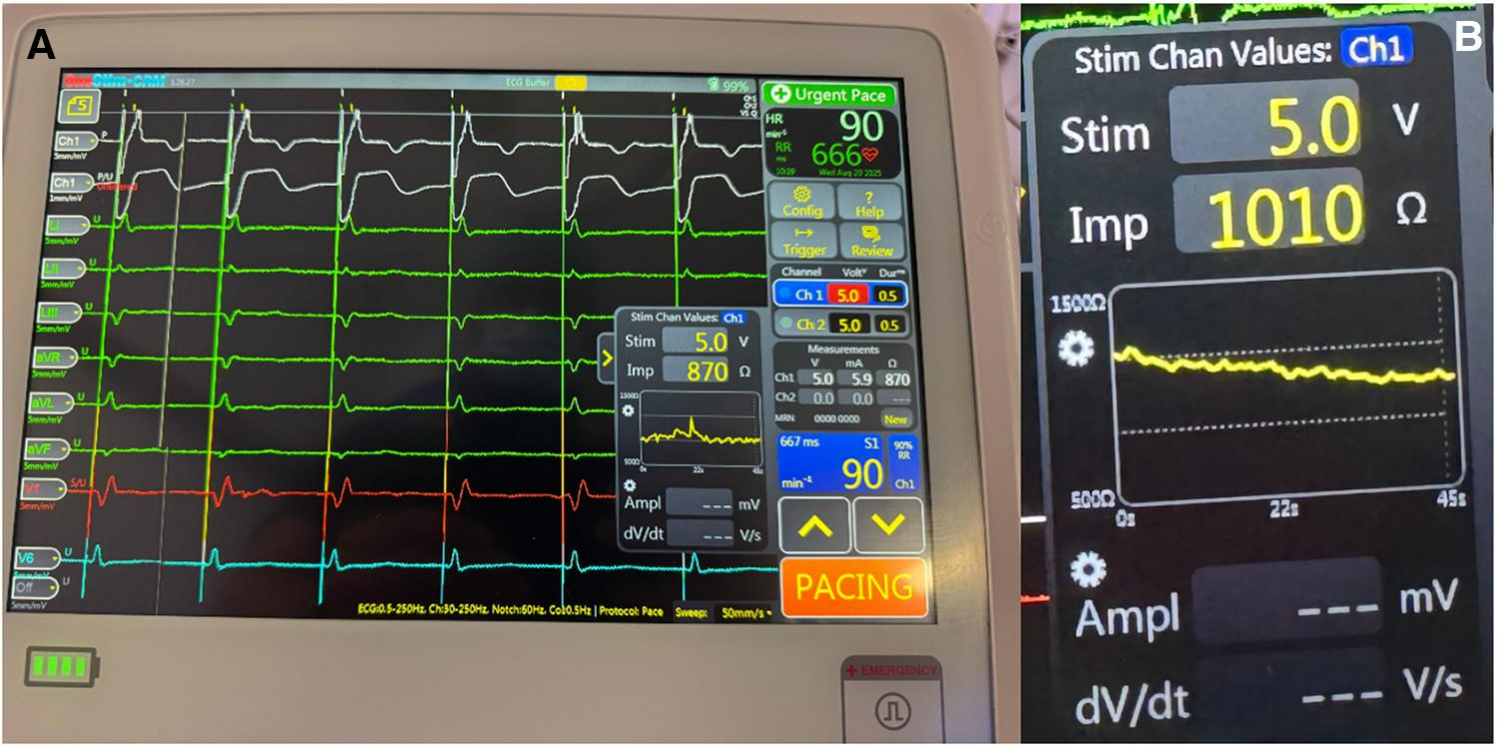
Continuous monitoring during septal penetration with the lumenless lead, final position (panel *A*); impedance monitor (panel *B*) performed with the new rotatable connector by Medtronic © and the Micropace © Onestim cardiac stimulator.

**Figure 5:**
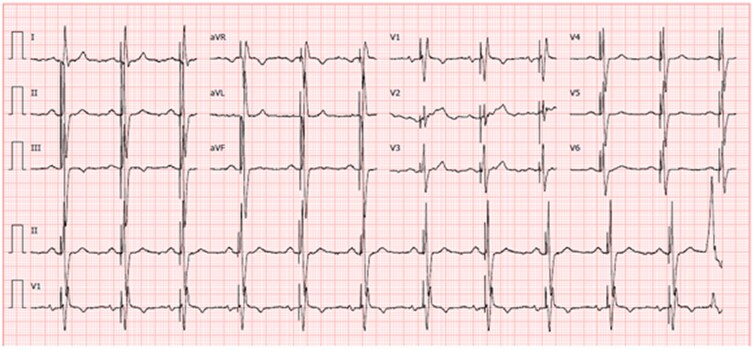
12-lead surface ECG with unipolar ventricular pacing confirming conduction system capture QRS width 112 ms and broad R′ in lead V1.

Procedure: Under local anaesthesia, venous access was obtained via the left subclavian vein. A C315HIS sheath was advanced into the right atrium and positioned at the ventricular septum region under fluoroscopic guidance. A Medtronic 3830 (SelectSecure™) lumenless lead was connected to the Micropace® Onestim cardiac stimulator through a rotatable connector (*Figure 2*), which allowed simultaneous pacing during rotational advancement. After confirmation of adequate lead positioning (*Figure 3*), this configuration permitted continuous monitoring of electrograms, pacing impedance, and current of injury (COI) on a beat-to-beat basis while the lead penetrated the interventricular septum (*Figure 4*). Criteria for successful CSP were met as seen in *Figure 4* panel A, including selective narrow QRS morphology (QRS width 112 ms), left ventricular activation time 62 ms, V6R to V1R 45 ms, and stable pacing parameters (ventricular sensing of 7.5 mV and threshold 1.3 V at 0.4 ms - *Table 1*). Final ECG was obtained—*Figure 5*. The total fluoroscopy time was 4:19 min, and the procedure was completed within 50 min. No complications occurred. The patient was discharged on the same day, with stable device parameters confirmed at 1-week follow-up. Final fluoroscopic position can be seen in Supplementary material online, *Figure S6*.

**Table 1:** Procedural and device parameters

Parameter	Baseline	During Septal penetration	Post-fixation	Follow-up
Sensing (mV)	6.7	Not evaluated	7.5	11.5
Pacing threshold (V @ 0.4 ms)	1.75	Not evaluated	1.3	0.9
Impedance (Ω)	∼900	As shown in *Figure 4* (≈900 Ω)	823	700–800

During penetration, real-time impedance and COI were continuously evaluated via the rotatable connector system as demonstrated in *Figure 4*.

## Discussion

This case highlights the potential advantages of using a rotatable connector to enable simultaneous pacing during lumenless lead deployment for CSP. Traditionally, operators rely on intermittent pacing, the occasional fixation beat, and impedance checks during septal penetration, which may increase the procedure complexity and fluoroscopy exposure.^3^ The ability to monitor COI and impedance in real-time provides immediate feedback on tissue engagement and conduction capture.

The connector used in this case (5944RL) is a commercially available, food and drug administration-approved, and conformité européenne-marked accessory. It was used in a sterile setup, with electrical continuity ensured through secure fixation and handling by the operating physician. The connector was stabilized externally to prevent contamination or torque transmission.

In the multicenter MELOS trial, mean fluoroscopy time was approximately 9 min,^1^ and in other trials with operators with more than 50 cases performed, total procedure time was around 80 min.^4^ In our case, both were shorter (4:19 min and 50 min, respectively) despite being performed by an operator with less than 50 CSP cases experience, which highlights procedural simplicity.

When continuous pacing during deployment is not possible, operators must rely on intermittent pacing or fluoroscopy to detect septal perforation or inadequate fixation. Potential complications include lead perforation, loss of conduction capture, or unstable thresholds, and such cases of inadvertent septal perforation during CSP were already reported.^5^ Real-time pacing and impedance feedback may help anticipate such issues by providing early warning of abrupt impedance or COI changes.

To our knowledge, this is the first reported clinical use of this technique, and while procedural parameters are documented in this article, systematic archiving of raw device interrogation data was not performed for this initial case. Further studies are required to assess reproducibility, long-term stability, and whether sustained pacing during fixation reduces procedural complexity or complication risk, but our case suggests it may represent a valuable adjunct in CSP practice.

## Lead author biography


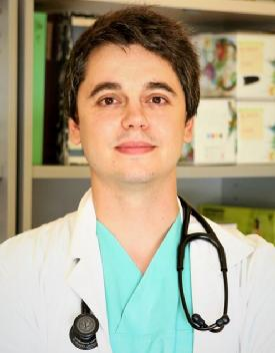


Fernando Montenegro Sá, MD, MSc, is a cardiologist from Porto, Portugal. He is an EHRA-certified Cardiac Devices Specialist and is the current head of the Arrhythmia unit for the ULSM - Hospital Pedro Hispano. In 2020, he joined the editorial board of the Portuguese Cardiology Journal. From 2021 to 2023, he was the Portuguese Young Cardiology Council coordinator, and since 2025, he has been a member of the national Portuguese Cardiology Society board. After graduating with a master’s in medical informatics, he works as a DARWIN-EU data partner and focuses on medical uses of smartwearable devices.

## Supplementary Material

ytag038_Supplementary_Data

## Data Availability

The data underlying this article are available in the article and in its online Supplementary material.
