# Supplementary figures and images for: Conduction system pacing using a rotatable connector enabling continuous pacing during lumenless lead deployment: a case report
Source: Eur Heart J Case Rep. 2026 Feb 3;10(2):ytag038. doi: 10.1093/ehjcr/ytag038 (PMC12908082; doi:10.1093/ehjcr/ytag038)

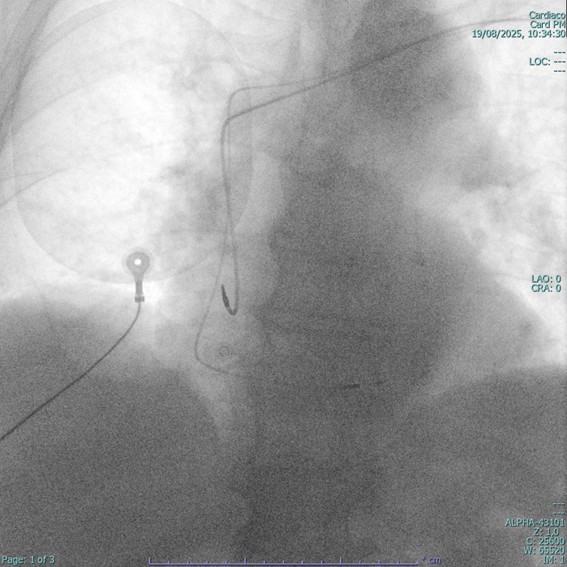

Supplement: ytag038_Supplementary_Data [file ytag038_supplementary_data.zip › Figure6.jpg]
